# Cellulose defibrillation and functionalization by plasma in liquid treatment

**DOI:** 10.1038/s41598-018-33687-2

**Published:** 2018-10-19

**Authors:** Sorin Vizireanu, Denis Mihaela Panaitescu, Cristian Andi Nicolae, Adriana Nicoleta Frone, Ioana Chiulan, Maria Daniela Ionita, Veronica Satulu, Lavinia Gabriela Carpen, Simona Petrescu, Ruxandra Birjega, Gheorghe Dinescu

**Affiliations:** 1 National Institute for Laser, Plasma and Radiation Physics, Atomistilor 409, Magurele, Bucharest, Ilfov 077125 Romania; 2 Polymer Department, National Institute for Research and Development in Chemistry and Petrochemistry, 202 Spl. Independentei, Bucharest, 060021 Romania; 3 Institute of Physical Chemistry “Ilie Murgulescu”, Romanian Academy of Sciences, 202 Spl. Independentei, Bucharest, 060021 Romania

## Abstract

Submerged liquid plasma (SLP) is a new and promising method to modify powder materials. Up to now, this technique has been mostly applied to carbonaceous materials, however, SLP shows great potential as a low-cost and environmental-friendly method to modify cellulose. In this work we demonstrate the modification of microcrystalline cellulose (MCC) by applying the SLP combined with ultrasonication treatments. The plasma generated either in an inert (argon) or reactive (argon: oxygen or argon:nitrogen) gas was used in MCC dispersions in water or acetonitrile:water mixtures. An enhanced defibrillation of MCC has been observed following the application of SLP. Furthermore, X-ray photoelectron spectroscopy and Fourier transform infrared spectroscopy have been applied to investigate the surface functionalization of MCC with oxygen or nitrogen moieties. Depending on the plasma treatment applied, poly (3-hydroxybutyrate) composites fabricated with the plasma modified cellulose fibers showed better thermal stability and mechanical properties than pristine PHB. This submerged liquid plasma processing method offers a unique approach for the activation of cellulose for defibrillation and functionalization, aiming towards an improved reinforcing ability of biopolymers.

## Introduction

The most widespread polymer on the earth, i.e. cellulose, may be used in a large range of applications, from food to medical devices^1–6^. Special attention is given to cellulose nanofibers as reinforcements in polymers to improve their properties^2–7^. Cellulose can be extracted from a multitude of sources (wood, plants, algae) or from cellulosic wastes by using combined mechanical, chemical or biological methods^2^. Acid hydrolysis using sulfuric, phosphoric or hydrochloric acid is one of the most used methods to obtain cellulose nanocrystals. The release of the cellulose crystals is determined by the penetration of the acid through the amorphous and defect zones of cellulose which leads to the split of cellulose particles. Enzymatic or acid hydrolysis and mechanical refining are often used as pretreatments to activate cellulose and to induce a more rapid disintegration^8^. Generally, the procedures to prepare cellulose nanofibers from a cellulosic source are laborious and expensive and they may obscure the extraordinary benefits of cellulose by using or disposing dangerous chemicals. Intensive research is devoted to design new, eco-friendly and industrially applicable methods for the preparation of cellulose nanofibers and their functionalization.

Ultrasound technology is considered a green approach with high potential in the defibrillation of cellulose^9,10^. Both physical (shear forces, surface erosion, cavitation) and chemical (oxidizing radicals) mechanisms are involved in this process. It is worth to mention that the energy provided by cavitation is within the hydrogen bond energy levels and may induce both defibrillation and reorganization in the amorphous phase of cellulose^11^. Earlier works have reported on the effect of high power ultrasonication in the release of cellulose nanofibers from microcrystalline cellulose^9^ or from microalgae^10^.

Moreover, plasma treatment is intensively studied to improve the surface properties of cellulose films or sheets^12–16^. In particular, the oxidative plasma treatment and grafting of L-lactide onto cellulose films led to an increased interfacial compatibility in polymer-cellulose composites^16^. Similarly, bacterial cellulose membranes modified by nitrogen-containing plasma showed enhanced cell adhesion and viability^13^. All reported treatments were applied to cellulose in the form of films, sheets or membranes^12–16^ as the plasma treatment of cellulose powders or fibers in vacuum or open atmosphere is a difficult task, affected by adverse phenomena such as suction, spreading, or limited contact. Such phenomena are avoided when the powders are dispersed in liquids. Submerged liquid plasma (SLP) is a recent research topic and a promising route to modify powder materials and nanomaterials. This procedure is environmentally friendly and has additional benefits related to the use of low temperatures during treatment, relatively simple installations (without vacuum components) and low operating cost. Various liquid media or a wide variety of reactive gases may be used in the discharge. Furthermore, the reactive species which appear at the liquid/plasma/particles interface^17,18^ may initiate specific reactions and may contribute to bonds breakage on the cellulose surface. Therefore, new properties may be generated by SLP to the liquid dispersions of cellulose. Although promising, the SLP technique was mostly applied to carbonaceous dispersions^19,20^ and only few works reported on its application to cellulose^21,22^.

In this paper, the treatment of cellulose by SLP and ultrasounds is proposed as a simple, cheap, and reproducible method to defibrillate and functionalize the surface of cellulose and as an environmentally friendly alternative to classical chemical routes. To the best of our knowledge this is the first study on the influence of submerged liquid plasma treatment on the defibrillation of cellulose and the increase of ultrasound treatment efficiency. Here, a plasma torch source^23^, initially designed for atmospheric pressure was completely immersed in the dispersions of microcrystalline cellulose (MCC) in water or acetonitrile (ACN)-water mixtures. Both inert and reactive gases were used for the treatment of MCC. The changes induced on the surface of cellulose by the combined action of plasma and ultrasounds were highlighted by X-ray photoelectron spectroscopy (XPS), Fourier transform infrared spectroscopy (FTIR), X-ray diffraction (XRD), and thermogravimetric analysis (TGA). The morphological changes were assessed by scanning electron microscopy (SEM). Plasma and ultrasounds modified celluloses were used as reinforcements in poly (3-hydroxybutyrate) (PHB). PHB is a biopolyester obtained by microbial synthesis which shows similar mechanical and barrier properties to several commodity plastics. This biopolyester is of great interest as the modified celluloses may improve its thermal and mechanical properties for medical and engineering applications^24,25^.

## Materials and Methods

### Materials

Microcrystalline cellulose (MCC) with 20 µm mean particle size was purchased from Sigma Aldrich (USA). MCC was obtained by acid hydrolysis of cotton linters. PHB type P304 from Biomer (Germany) was used to prepare the composites. This PHB grade may be processed by melting using laboratory equipment for common thermoplastics and may withstand a wide range of temperatures, between −30 °C and 120 °C. The tensile strength (according to ISO 527, 50 mm/min) of PHB P304, provided by the producer, is 28 MPa. Acetonitrile 99% was purchased from Fluka (Switzerland) and used as received.

### Plasma treatment of cellulose suspensions

**Figure 1:**
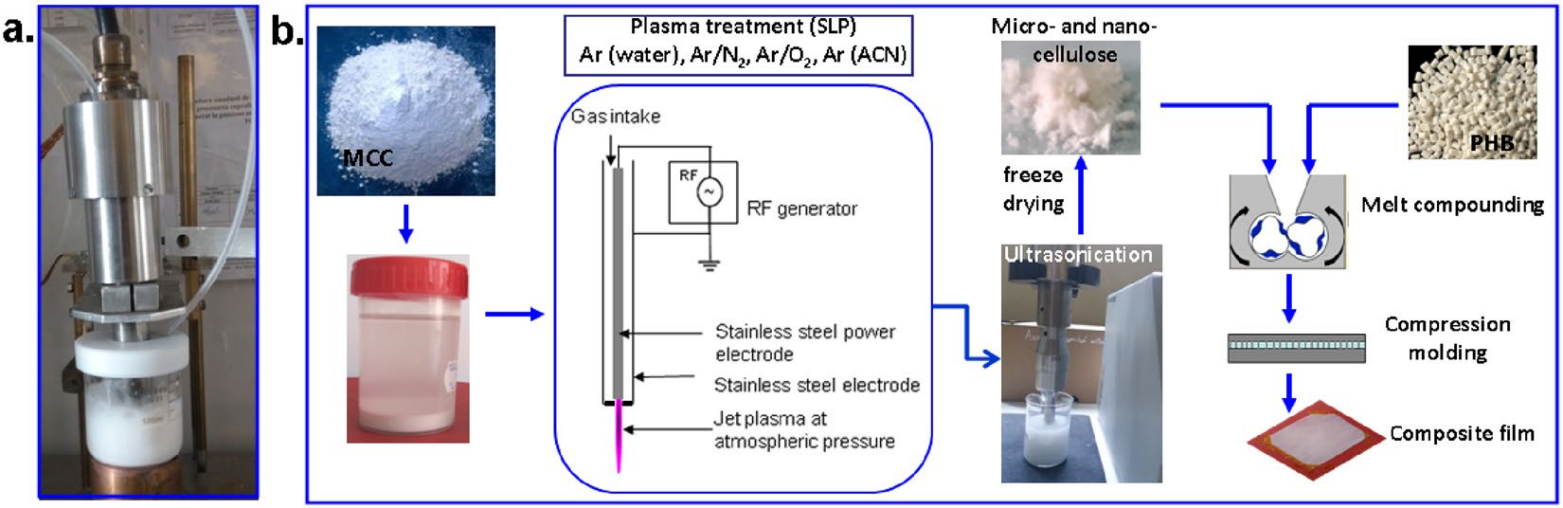
(**a**) Image during the treatment with plasma torch immersed in the cellulose suspension; (**b**) Schematic representation of the experimental setup.

The cellulose suspensions (5 wt%) were prepared by dispersing 10 g MCC in 0.2 L of bi-distilled water by ultrasonication for 1 h. An ultrasonic bath Elmasonic S 15 H (Elma, Germany) was used for this purpose and a power density of 0.12 W/mL. The concentration of MCC suspension in water was chosen based on earlier reports^26,27^. MCC suspensions with a concentration in the range 2–10% are frequently used for surface modifications^26–29^. In general, higher particle concentrations result in an increased collision frequency and, possibly, to agglomerations while lower particle concentrations lead to fewer reactive sites in suspension. Therefore, a medium concentration was preferred to ensure optimum conditions. The plasma treatment of MCC suspensions was carried out using a plasma torch source, designed to work at atmospheric pressure in open air^23^. In a previous work, this source was successfully used for the functionalization of a graphene suspension^20^. In this study, the plasma jet was ignited in open air (5000 sccm argon, 150 W) and immersed in 80 ml MCC suspension for 30 or 60 min (Fig. 1a). In some experiments, additional reactive gases (oxygen or nitrogen) were introduced in the main argon flow. Furthermore, ACN was mixed in the water suspension of MCC to induce new functional groups on the cellulose surface. The sample ID, labeled from the reactive agent and the experimental conditions, are presented in Table 1.

**Table 1:** Conditions for plasma torch treatment of MCC.

Samples ID	Ar flow (sccm)	Reactive gas/liquid	RF power (W)	Treatment duration (min)
MCC Ar	5000		150	30 and 60
MCC Ar/N_2_	5000	N_2_ (500 sccm)	150	30 and 60
MCC Ar/O_2_	5000	O_2_ (250 and 500 sccm)	150	30
MCC Ar-CAN	5000	ACN 30% in water	150	30

### Ultrasonication and freeze-drying of untreated and plasma treated MCC

MCC suspensions in distilled water, containing untreated or plasma treated cellulose (0.2 L), were ultrasonicated for 30 min using a 20 kHz Vibra Cell VC 505 ultrasonic processor (Sonics, USA), with a maximum power of 500 W and a titanium alloy probe of 19 mm. The working power was set to 20% of the maximum power, corresponding to a power input of 100 W, in order to avoid contamination of the cellulose samples with metal nanoparticles; the power density was 0.5 W/mL. Ultrasonicated plasma treated MCC samples were lyophilized using a FreeZone 2.5 L Benchtop Freeze Dry System (Labconco, USA). The schematic representation of the experimental setup is shown in Fig. 1b.

### Preparation of PHB composites with untreated and plasma treated MCC

PHB pellets and untreated or plasma treated MCC were further dried in vacuum ovens at 60 °C for 2 h and 50 °C for 4 h. MCC (2 wt%) was added in PHB using a Brabender LabStation (Duisburg, Germany) with a mixing chamber of 30 cm^3^. Melt mixing was carried out at 165 °C for 10 min at a rotor speed of 50 min^−1^. Films with a thickness of 200–250 µm were used for mechanical characterization and 1 mm thick sheets for morphological investigation. Both sets of samples were obtained by compression molding using an electrically heated press (P200E, Dr. Collin, Germany) at 175 °C, with 120 s preheating (5 bar), 75 s under pressure (100 bar), and cooling for 1 min in a cooling cassette.

### Characterization

#### Morphological investigation by SEM

Pristine MCC as well as plasma treated and ultrasonicated MCC samples were dispersed in distilled water and one drop was deposited on double-sided conductive tapes fixed on aluminum stubs. After drying in a vacuum oven at 50 °C for one hour, the samples were sputter-coated with a thin layer of gold (10 nm) using a coating unit Cressington sputter coater 108 auto with thickness monitors (Cressington, UK). SEM images of the surface coated samples were captured with a FEI Quanta 3D FEG dual beam instrument (FEI, USA), operated in high vacuum at 5–20 kV accelerating voltage. The compression molded sheets of PHB – MCC composites were fractured in liquid nitrogen and fixed vertically on silicon substrates with double-sided conductive tapes, then sputter-coated with a thin layer of gold and analyzed by SEM using the same instruments.

#### Chemical characterization by XPS and FTIR

Several drops of untreated and plasma treated cellulose suspensions were deposited on silicon wafers and dried at room temperature for a couple of days. The resulted cellulose films were investigated by X-ray photoelectron spectroscopy using ESCALAB™ XI+ spectrometer (Thermo Scientific, USA) with a monochromatic Al Kα source at 1486.6 eV. The XPS spectra were recorded as survey spectra (step of 1 eV) at a pass energy of 100 eV and as high-resolution scans in the C 1 s, O 1 s and N 1 s regions, step of 0.1 eV, for 10 scans and the pass energy of 20 eV. The XPS spectrum of MCC reference was calibrated for Au 4f 7/2 binding energy at 84.0 ± 0.1 eV. The Au reference (5 nm thick) was deposited by magnetron sputtering. The untreated and plasma treated cellulose films were also analyzed by FTIR using Tensor 37 spectrometer with ATR setup from Bruker Optics (USA). The spectra were collected at room temperature, in the spectral range 4000 to 400 cm^−1^, at a resolution of 4 cm^−1^ using 16 scans. The background spectrum was collected before measuring every sample. Spectra were baseline-corrected using the OPUS spectroscopy software (Bruker Optics, USA) and normalized with respect to the absorption at 2895 cm^−1^, corresponding to the C−H stretching vibration^30^.

#### Thermal characterization

Thermogravimetric analysis was used to characterize the thermal stability of MCC samples before and after the plasma treatment and the thermal behavior of PHB composites with untreated and plasma treated cellulose. TGA was carried out on a TA-Q5000 V3.13 system (TA Instruments Inc., USA) using nitrogen as the purge gas at a flow rate of 40 mL/min. The samples were heated from 25 °C to 700 °C at a heating rate of 10 °C/min.

#### Mechanical characterization

The tensile properties of PHB composites were measured at room temperature using an Instron 3382 universal testing machine (USA) with a crosshead speed of 2 mm/min. Five specimens according to ISO 527 were tested for each composite.

#### X-ray diffraction

XRD measurements were carried out at room temperature on the films of plasma treated and ultrasonicated MCC deposited on Si wafers using a PANalytical X’Pert PRO MPD X-ray diffractometer. Incident X-ray radiation was produced from a line focused Cu X-ray tube, with a Kα wavelength of 1.5418 Å. A curved graphite monochromator and a variable divergence slit working in the fixed irradiated area mode were placed in the diffracted beam. Measurements were done in the 2θ range from 10° to 30°.

## Results and Discussion

### Effect of plasma exposure on the thermal stability of cellulose

**Figure 2:**
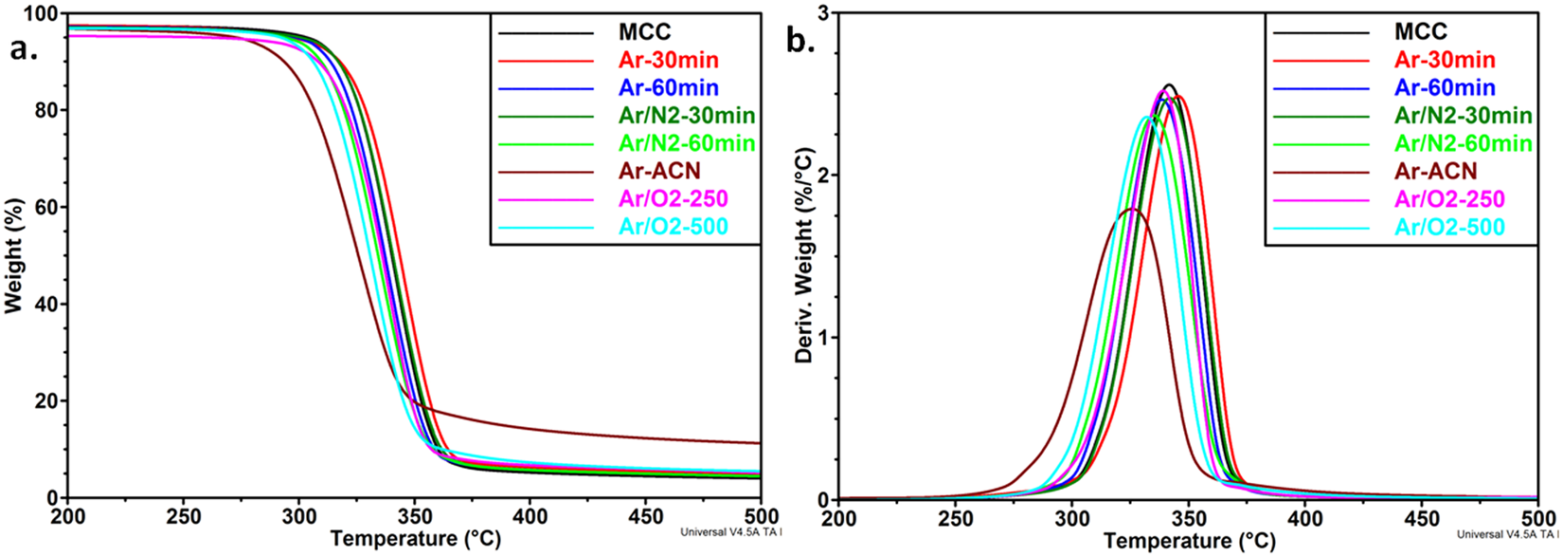
TGA (**a**) and DTG (**b**) curves for plasma treated cellulose samples.

The treatment of cellulose suspensions using cold plasma allows a gentle modification of the cellulose surface at a temperature near or slightly above the ambient temperature. The reactive species, radicals, charged particles or atomic excites species, generated during this treatment, may affect the thermal stability of cellulose. Therefore, the influence of different reactive gases, treatment durations or gas flow rates was investigated by thermogravimetric analysis. TGA and derivative curves are shown in Fig. 2a,b. One single weight drop was noticed for all the samples between 300 and 360 °C due to the depolymerization and dehydration, followed by the destruction of the glucopyranosyl units^31^. However, the thermal stability was influenced in different ways by the nature of the gas and duration. For comparison, the onset temperature of thermal degradation (*T*_*on*_) and the temperature of the maximum degradation rate (*T*_*max*_) of all samples prepared in different plasma conditions are given in Table S1 of the Supplementary Information. Ar and Ar/N_2_ mixture slightly increased the *T*_*max*_ of MCC when the plasma treatment duration was 30 min. In contrast, a higher duration (one hour) decreased the *T*_*max*_ value with 7–8 °C. Similarly, a low O_2_ flow rate (250 sccm) didn’t change the thermal stability of cellulose, whereas a higher O_2_ flow rate (500 sccm) decreased the *T*_*max*_ value with 9 °C. Therefore, the intensification of plasma treatment conditions and the use of reactive gases/agents have as result a decrease of the thermal stability of cellulose. A similar decrease of the thermal stability was reported after the dielectric barrier discharge (DBD) plasma treatment of banana fibers^32^ and similar onset of cellulose depolymerization (about 330 °C) was reported in the case of untreated and low-temperature plasma treated cotton fibers^33^.

In the case of Ar plasma applied to a MCC suspended in ACN - water mixture, the *T*_*on*_ value was decreased with 20 °C (Fig. 2). The value of the residue at 500 °C (*R*_500_), i.e. 12.3% for Ar-ACN and about 5% for untreated MCC and MCC treated in all the other conditions indicates that some reactions (grafting, cross-linking) were involved in the case of ACN liquid plasma treatment. A similar increase of the residue was reported for plasma treated Quiscal fibers and explained by the cross-linking of polymer chains due to the plasma exposure^34^. Moreover, the graft polymerization of butyl acrylate on cellulosic pine needles using microwaves or on H. sabdariffa fibers by a chemical method resulted in an almost two-times increase of the residue^35,36^. Senthilnathan *et al*. have reported that low-molecular nitrogen polymers were formed by the immersion of a high-voltage discharge between a tungsten needle and Si wafer (used as a planar ground electrode) in the acetonitrile solution^17^. Therefore, the decrease of the T_max_ value of MCC in some plasma treatment conditions may be an indication of the effective surface modification of cellulose. To verify this hypothesis, the changes in the chemical composition after the plasma treatments were studied by XPS. For subsequent comparison only 30 min plasma exposure was selected.

### Surface chemical properties of plasma treated cellulose

**Figure 3:**
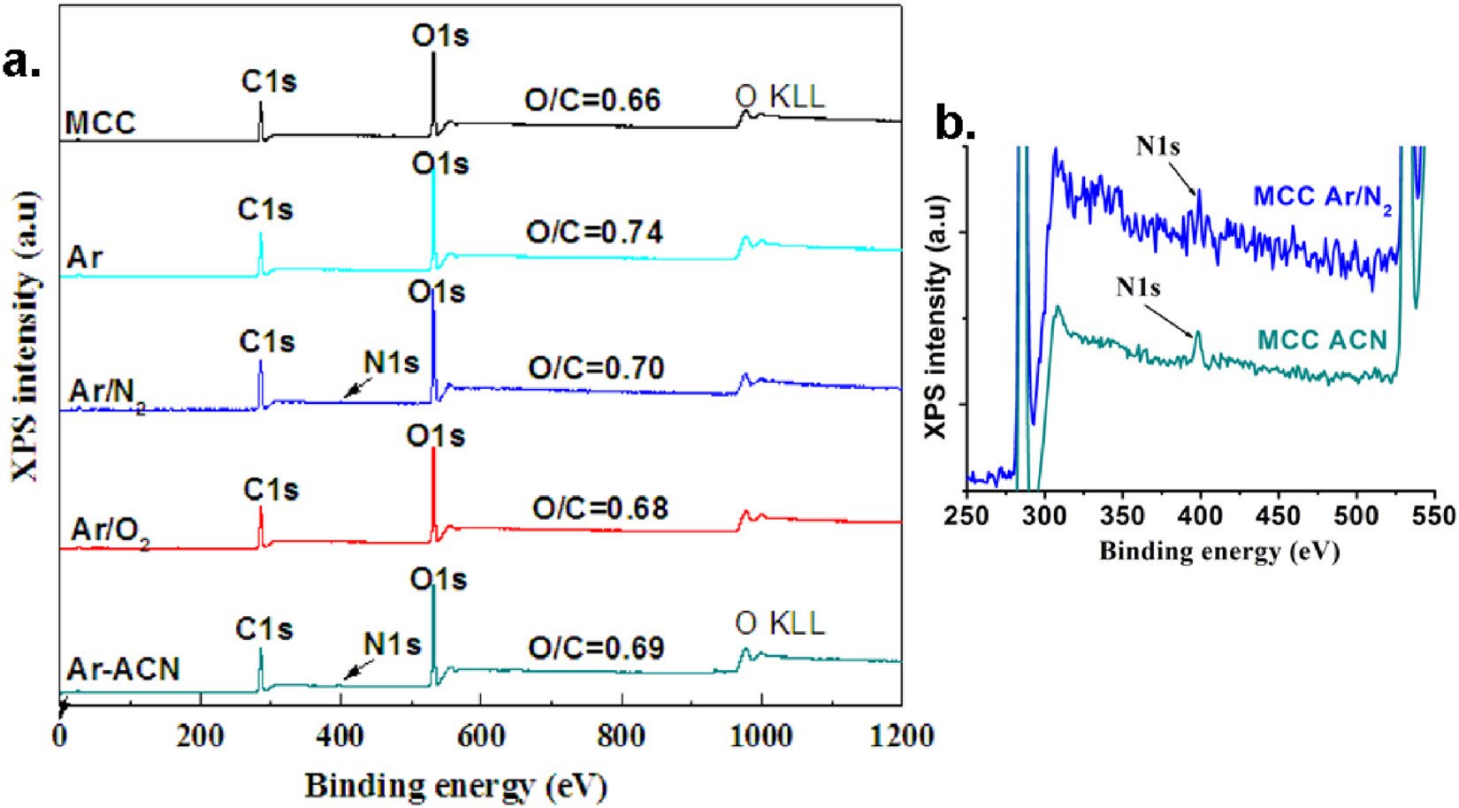
XPS survey spectra of MCC and plasma treated cellulose (**a**) zoom of the spectra showing N1s peak (**b**).

The XPS survey spectra (Fig. 3a) indicates that carbon and oxygen are the main elements on the surface of cellulose before and after treatments. N1s peak was detected in the case of MCC Ar/N_2_ and MCC Ar-ACN (Fig. 3b), showing the insertion of a few percent of nitrogen after these treatments. Negligible amounts of metal contaminants, either coming from the discharge electrodes (molybdenum) or from the calibration procedure (gold) were also noticed. The relative atomic percentage of elements depends on the reactive agents used in the treatment as observed from Table 2.

**Table 2:** Atomic concentration of carbon and oxygen in plasma treated cellulose determined by XPS.

Samples	MCC	MCC Ar	MCC Ar/N_2_	MCC Ar/O_2_	MCC Ar-ACN
C 1 s (%)	60.3	57.5	58.6	59.6	58.5
O 1s (%)	39.7	42.5	40.9	40.4	40.2
N 1s (%)	0.0	0.0	0.5	0.0	1.3

The O/C ratio was calculated to assess the oxidation level following the treatments. A value of 0.66 was obtained for untreated MCC, quite different from 0.83, the theoretical ratio for pure cellulose, due to impurities and contamination^13,37^. An increase of O/C ratio was observed after the plasma treatments, associated to the oxidation and removal of the impurities. Indeed, Vander Wielen *et al*.^12^ observed an increase of O/C ratio from 0.78 for untreated cellulose fibers (fully bleached softwood pulp) to 0.84 after the dielectric-barrier discharge treatment. This increase was explained by the oxidative reactions along with the surface cleaning due to the removal of contaminants, extractives and residual lignin. Previous work has shown an increase in surface reaction efficiency of nanocellulose films when UV/O_3_ pre-activation was conducted prior to the functionalization with aminosilane^38^. This was explained by the role of UV/O_3_ in removing the carbon-based compounds by the cleavage of organic bonds. Looking at the O/C ratio after plasma treatments (Fig. 3), it seems that SLP removed the contaminants and functionalized the cellulose surface at different rates depending on the conditions applied. The highest increase of O/C ratio was observed after Ar and Ar/N_2_ treatments (Fig. 3a) suggesting higher oxidation or cleaning effect. Nevertheless, all plasma torch treatments led to the chemical modification of MCC surface.

**Figure 4:**
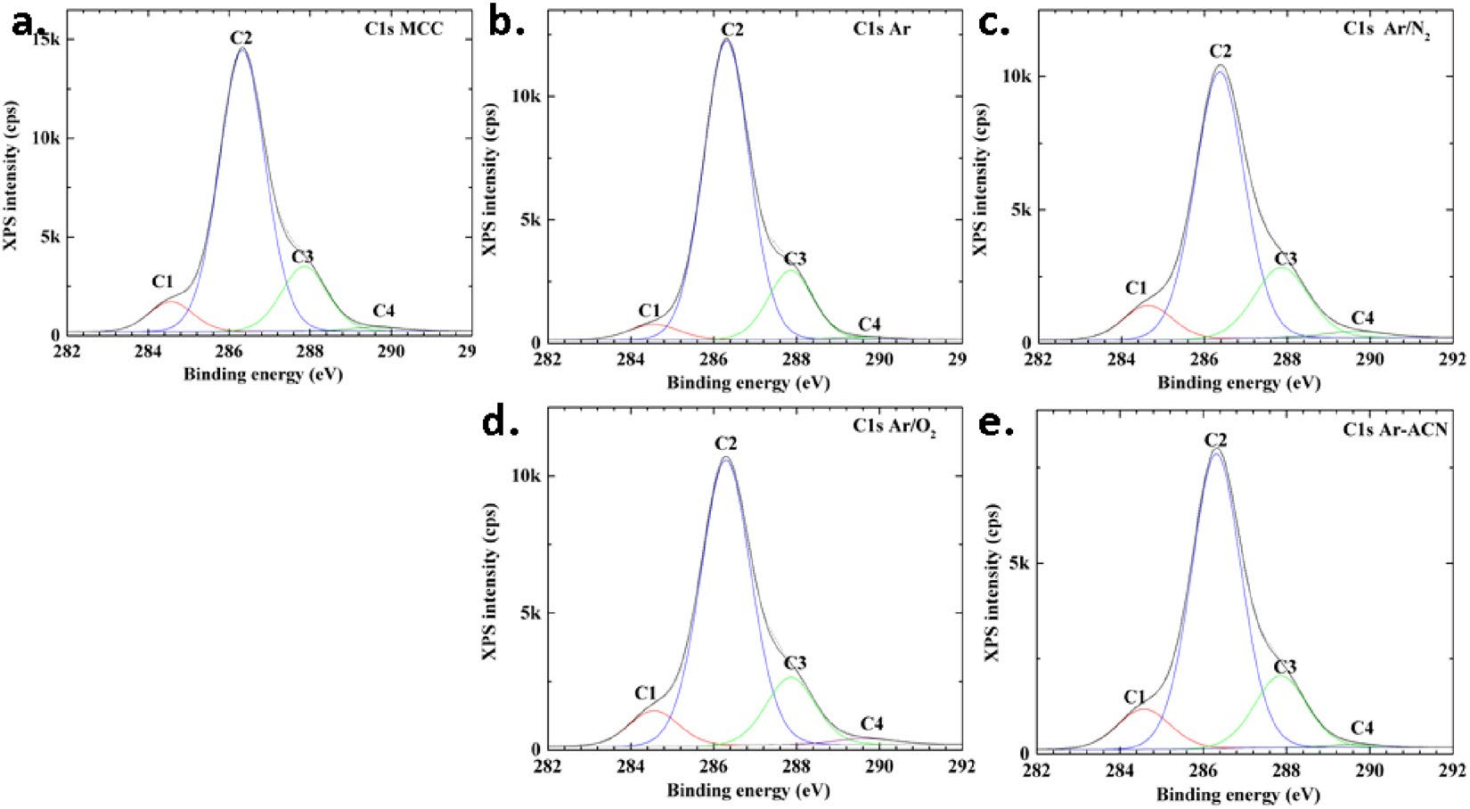
High resolution spectra of samples in the C1s region: (**a**) MCC (**b**) MCC Ar, (**c**) MCC Ar/N_2_, (**d**) MCC Ar/O_2_, and (**e**) MCC Ar-CAN.

For the identification of the bond type at the surface of cellulose, high resolution scans were carried out in the C1s and O1s regions. The high-resolution spectra and their deconvolutions are shown in Fig. 4a–e. The binding energies were calibrated based on the C1s peak at 286.3 eV, as found for the main peak of MCC (according to calibration by using Au 4 f peak) and all spectra were fitted for the same values of the binding energy.

**Figure 5:**
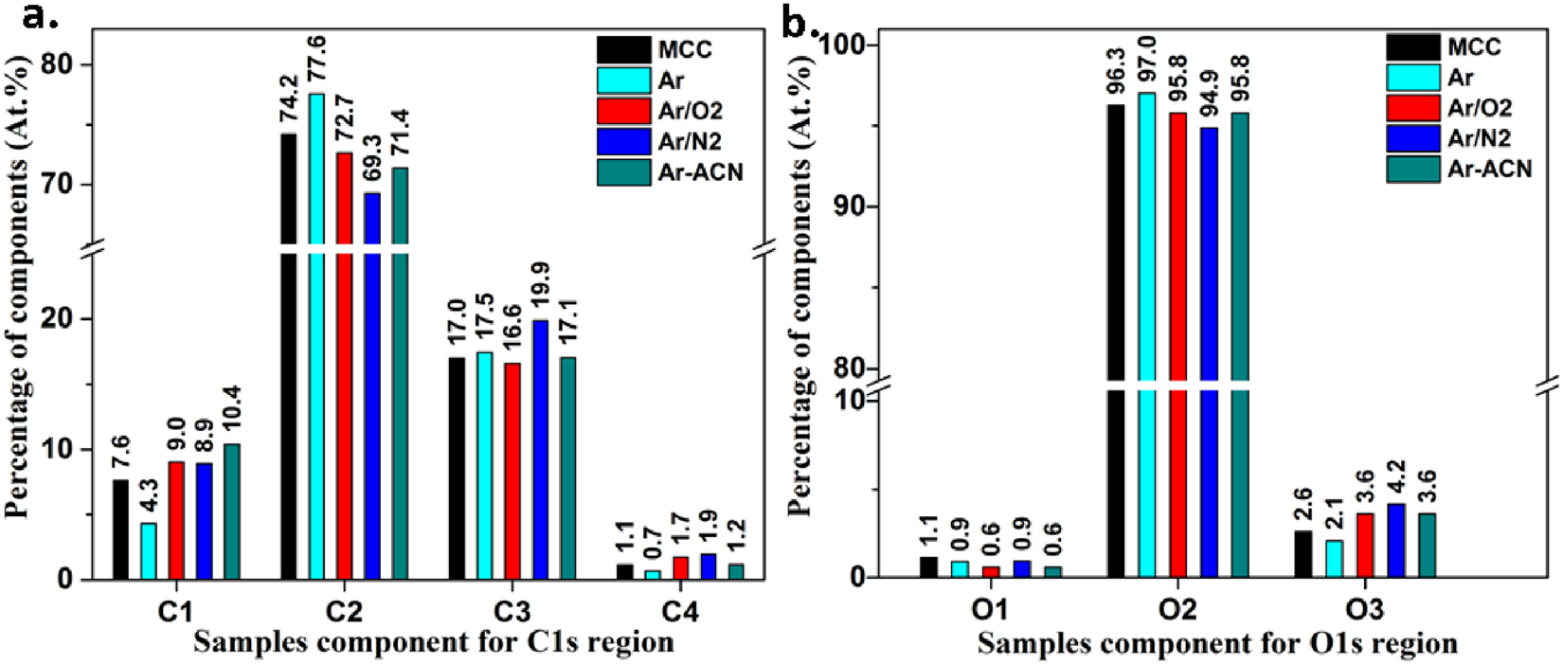
Atomic percentage of each component in (**a**) carbon region C1–C4 and (**b**) oxygen region O1-O3.

The C1s region was fitted with four peaks which were assigned to: C1 at 284.6 eV corresponding to a carbon atom bonded to other carbon or hydrogen atoms (C-C, C-H), C2 at 286.3 eV assigned to a C bonded to a single O in alcohol or ether groups (C-O), C3 at 288.0 eV which corresponds to a C bonded to two O atoms (O-C-O) or to a carbon atom double-bonded to an oxygen atom (C = O) whereas C4 at 289.7 eV represents C bonded to two O atoms, with one of them a carbonyl O atom (O-C = O). The O1s region was fitted considering the binding energy at 531.0 eV (O1, double bond O in O = C), at 532.9 eV (O2, single bonded O in -O*-C, C-O, HO*-C), and 535.0 eV (O3, attributed to O-COH, to chemisorbed O, water or O-C-OR). The presence of C1 type carbon atoms in untreated and plasma treated MCC was attributed to impurities and contamination as reported in previous works^13,37,38^ and explains the low O/C value. However, the carbon composition C1/C2/C3/C4 changed after the treatments, as shown in Fig. 5a,b.

The percentage of C-C/C-H bonds (C1 component) is higher for all the treatments compared to untreated MCC except for Ar plasma treatment where the C1 proportion decreased. Plasma treatment in Ar led also to the highest C2 proportion showing an increase of C-O bonds from alcohols and ethers, characteristic to cellulose. It may be hypothesized that Ar treatment of MCC is more efficient in removing the impurities from cellulose surface rather than in oxidation. Indeed, the application of the dielectric barrier discharge to wheat straw resulted in an etching effect; the charged particles in the plasma degraded the polymers and impurities on the surface of wheat straw, with the O/C ratio increasing from 0.35 to 0.64 after only one minute of plasma treatment^39^. This is consistent with the removal of C-C bonds containing contaminants from the surface of MCC and the breakage of C-C chemical bonds leading to an increased proportion of C-O bonds characteristic to cellulose. One may conclude that the Ar plasma treatment has a complex effect, first of cleaning the surface of cellulose from contaminants and, second, of cellulose surface functionalization. In our previous work^22^ we also noticed that the filamentary DBD plasma in pure Ar was more efficient in cleaning the surface of nanocellulose (observed by a reduction in the C1 proportion in XPS spectra) with respect to the same treatment in Ar/O_2_ or Ar/N_2_ gas mixtures. We also observed a much greater effect of plasma torch compared to filamentary DBD in removing the impurities.

The treatment in Ar/O_2_ led to small changes in C1/C2/C3/C4 composition, however it was obvious the increase of C4 component, which corresponds to the highest oxidation state (from 1.1% to 1.7%). A similar trend was observed for the sample treated in Ar/N_2_ where the proportion of C3 and C4 carbon atoms was further increased. Moreover, a slight decrease of the O1 peak and an increase of the O3 peak were noticed in both cases, showing a higher COOH accumulation after Ar/O_2_ or Ar/N_2_ treatments as compared to the untreated MCC.

A different behavior was noticed for MCC Ar-ACN. No important change in C2/C3/C4 proportion compared to the untreated MCC was observed. The difference appeared in the insertion of nitrogen (1.3%) and the increase of the C-C bonds proportion from 7.6% for MCC to 10.4% for MCC Ar-ACN. Similarly, the treatment of a bacterial cellulose membrane in a plasma reactor fed with N_2_ led to a reduction of the O proportion with an increase of both C and N proportions^13^ due to the new groups on the surface of cellulose. We may presume that the insertion of both nitrogen and carbon was induced by the plasma treatment of MCC in the ACN - water mixture. This supposition is supported by previous experimental results which demonstrated the formation of unconventional nitrogen polymers by plasma submerged in ACN solution^17^. Therefore, plasma initiates the hydrogen detachment in ACN and the formation of highly reactive free-radicals (•CH_2_-C≡N) which may polymerize in the absence of a support^17^ or can be grafted on the surface of cellulose in the presence of MCC as is the case in our experiments. Therefore, the Ar plasma torch treatment in ACN – water mixture activated the cellulose surface and inserted C-N containing groups. Some differences were noticed for nanocellulose treated in Ar plasma (DBD, ACN – water mixture)^22^. Although both methods have as result the grafting of nitrogen containing groups on cellulose, different chemical changes were noticed: the presence of imines and increased COOH proportion were observed in the XPS spectra of nanocellulose (DBD plasma) and an increase of C-C/C-N proportion in the case of plasma torch treated MCC. These differences may be explained by the larger surface area of nanocellulose with respect to MCC and the different surface activity.

The analysis of the O1s spectra showed an increase of the O3 component in plasma treated MCC when additional reactive gases (Ar/O_2_ and Ar/N_2_ mixtures) or ACN were used in the cellulose suspensions. The increase of the O3 percentage indicates higher accumulation of COOH groups on the surface of MCC and increased oxidation due to the treatments. Accordingly, a decrease of the onset temperature of degradation (see Table S1 of the Supplementary Information) was noticed in the TGA thermograms for MCC treated by Ar-ACN and Ar/O_2_ (500 sccm).

**Figure 6:**
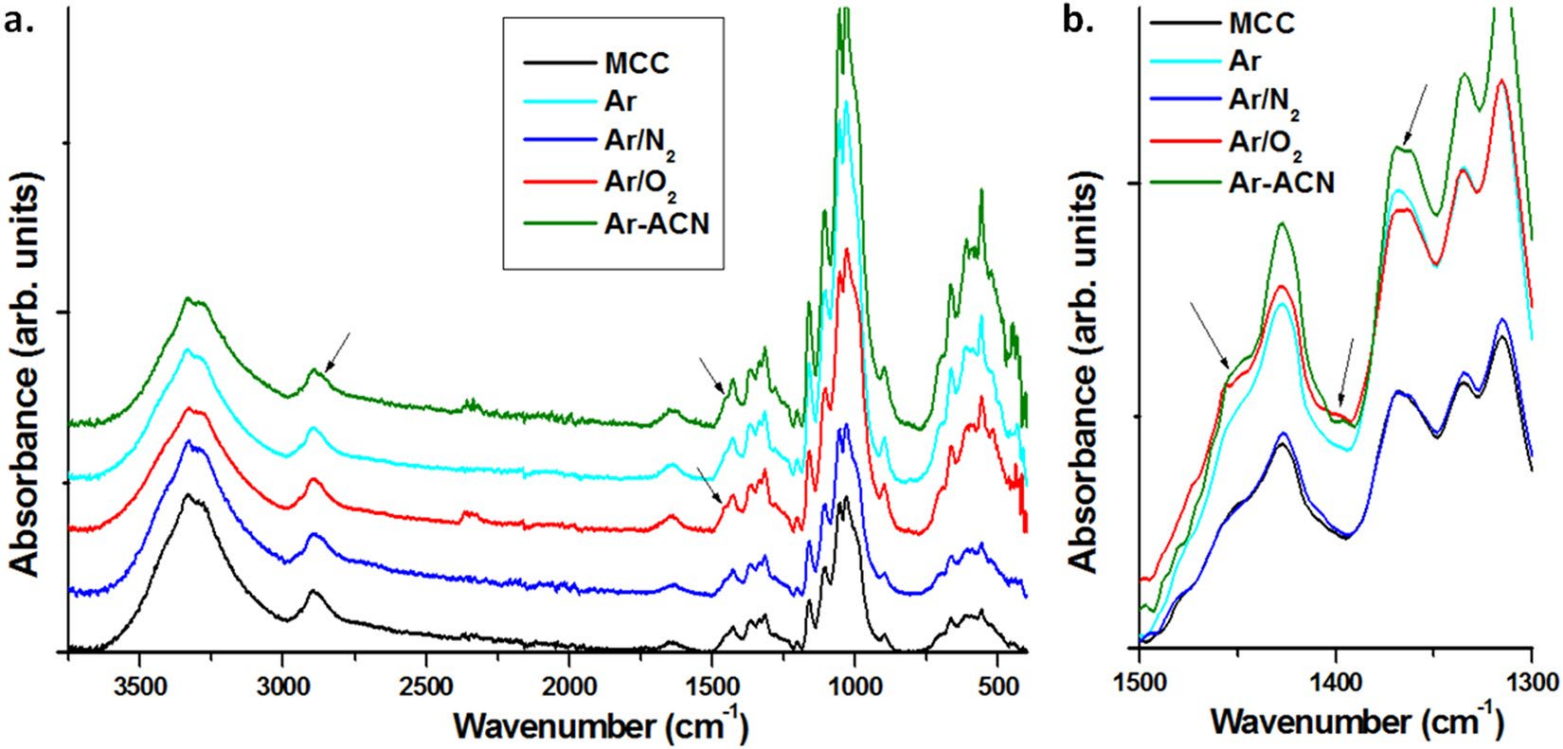
FTIR spectra of untreated and plasma treated MCC (**a**); detail – 1500–1300 cm^−1^ (**b**).

Furthermore, we carried out FTIR analysis on the same samples to gain more information on the surface groups (Fig. 6a).

Broadening of the peak assigned to aliphatic C–H stretching in Ar-ACN treated MCC to lower wavenumbers, i.e. 2850 cm^−1^ (untreated MCC) to 2810 cm^−1^ (Ar-ACN treated MCC) may indicate new O-CH_3_ or N-CH_3_ groups on the surface of cellulose, knowing that the CH_3_ symmetric stretching vibration is altered by the adjacent groups^40^. Several changes in the peaks at 1427 cm^−1^, 1370 cm^−1^ and 1315 cm^−1^, assigned to CH_2_ and CH deformation vibrations^41^, were observed after Ar-ACN and Ar/O_2_ plasma treatments (Fig. 6b): (i) new shoulders appeared at 1448–1456 cm^−1^ and 1396 cm^−1^ which may be assigned to C-N vibration in amines or C-H vibration in aldehydes, cetones or esters^40^; (ii) the bifurcation of the peak at 1370 cm^−1^ may also indicate changes in the interactions or environment. Similarly, the broadening of the peak at 1700 – 1600 cm^−1^ after Ar/O_2_ and Ar-ACN treatments indicates the presence of carbonyl and carboxyl groups overlapped with the OH bending of the absorbed water^42,43^. These chemical modifications of the cellulose surface are possible due to the reactive species (•OH, OH^−^, O_2_^−^, •NH_2_) which may appear in water after plasma immersion^44^. No important changes were noticed in the position of the bands characteristic to C-O-C asymmetric stretching and C-O stretching vibrations at 1160 and 1057 cm^−1^ ^41,45^, showing that the cellulose backbone was not significantly disturbed, in agreement with the small changes observed in the thermal behavior of MCC after the 30 min plasma treatments.

**Figure 7:**
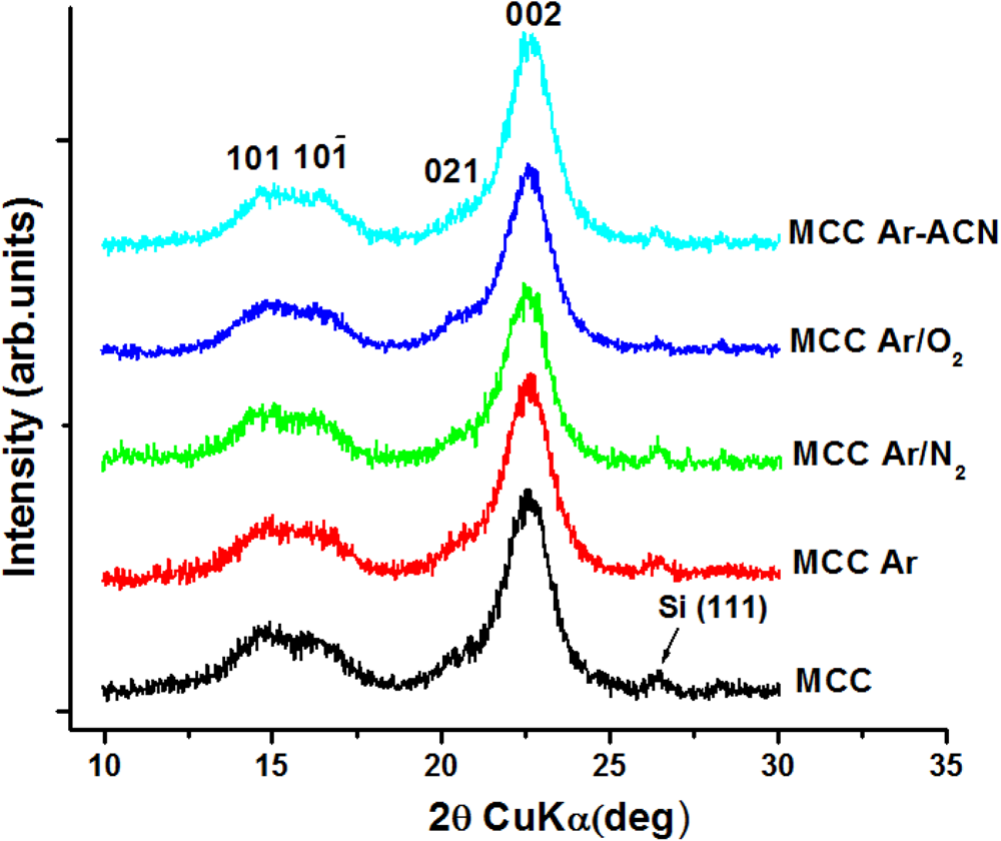
XRD patterns of MCC reference and plasma treated MCC samples.

The changes observed in the intensity of the peak at 897 cm^−1^ (antisymmetric out-of-plane ring stretch of amorphous cellulose)^46^ relative to that at 1427 cm^−1^, characteristic to the structural organization of cellulose suggest a small variation in the degree of order or crystallinity of MCC after the treatments. Therefore, XRD analysis was carried out to observe the influence of the treatments on the crystalline structure of cellulose. XRD patterns of untreated and plasma treated MCC are shown in Fig. 7. The cellulose samples were ultrasonicated before deposition on Si wafers.

All samples show the diffraction peaks characteristic to cellulose Iβ^9,47^. A small shoulder coming from the Si (111) planes of the substrate^48^ was also observed. The XRD crystallinity (*X*_*C*_), calculated as a ratio between the areas under the crystalline peaks and under the crystalline and amorphous peaks, did not significantly change after the plasma treatments (Table 3), showing that the crystalline structure is not altered by SLP, regardless the conditions. Similarly, the interplanar distance and the crystallite size were almost unchanged. We can assume that only the amorphous layers on the surface of cellulose were influenced mainly by the chemical and mechanical actions induced by SLP. Similar results were reported by Calvimontes *et al*.^49^ which observed no change in crystallinity for the films of regenerated cellulose treated in oxygen plasma for 480 s.

**Table 3:** XRD structural data for MCC before and after SLP treatments.

Samples	*Xc* (%)	*d*_002_ (nm)	*D*_002_ (nm)
MCC	84.3	0.393	5.37
MCC Ar	83.4	0.393	5.33
MCC Ar/N_2_	82.3	0.394	5.43
MCC Ar/O_2_	83.7	0.393	5.36
MCC Ar-CAN	83.6	0.392	5.34

*d*_002_ – the interplanar distances at the main peak calculated using the Bragg’s law.

*D*_002_ – the crystallite sizes at the main peak determined using the Scherrer equation ${\rm{D}}=\frac{0.9\,\lambda }{\beta \,cos{\rm{\Theta }}},$ where β is the full-width at half-maximum of the peak (in radians) and θ, the Bragg angle.

Correlating XPS and FTIR results, it can be assumed that the plasma treatments had different effects on cellulose, some conditions leading mostly to defibrillation and others to surface chemical modifications. SEM analysis was carried out to observe the morphological changes induced by SLP – ultrasonication treatments.

### SEM investigation of MCC before and after treatments

**Figure 8:**
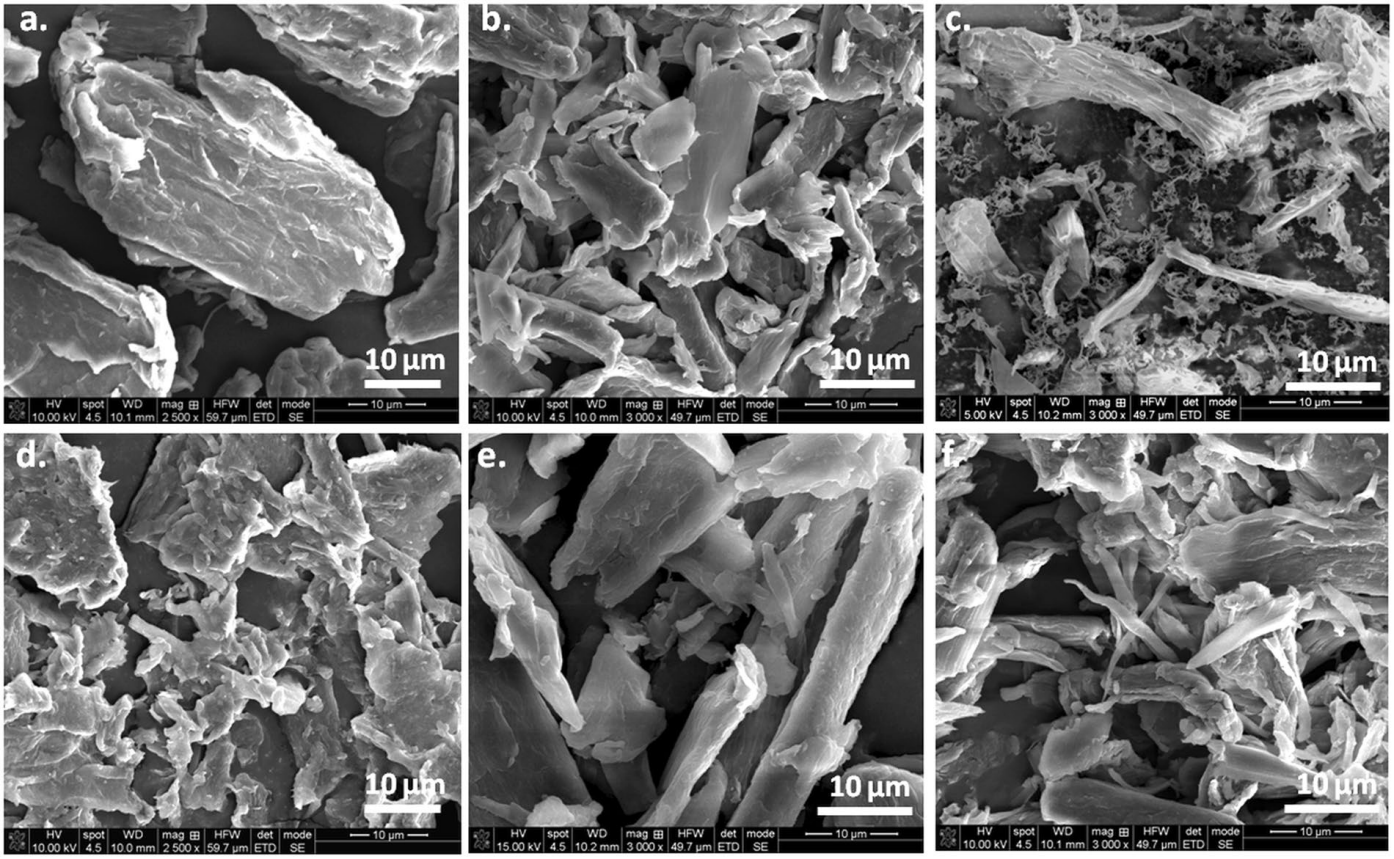
SEM images of untreated MCC (**a**), ultrasonicated MCC (**b**), and plasma treated MCC in Ar (**c**) Ar/N_2_ (**d**) Ar/O_2_ (**e**) and Ar-ACN (**f**).

SEM images of MCC samples before and after ultrasonic and plasma treatments are shown in Fig. 8a–f. Pristine MCC has micrometric size (Fig. 8a) and low aspect ratio (2–5), with lengths mostly between 20 and 80 µm and widths between 10 and 25 µm. After ultrasonic (Fig. 8b) and plasma - ultrasonic treatments (Fig. 8c–f), a size reduction of the MCC has been observed, depending on the treatment conditions. Ultrasounds caused the fibers breaking and defibrillation, with a small increase of the aspect ratio (3–7) compared to pristine MCC (Fig. 8b). After ultrasonic treatment, the widths of the fibers varied within a wide range, from 1 µm to 20 µm and the lengths rarely exceeded 30 µm. Previous reports have also shown that ultrasonication caused the fibers breaking^50^ and defibrillation^9^.

**Figure 9:**
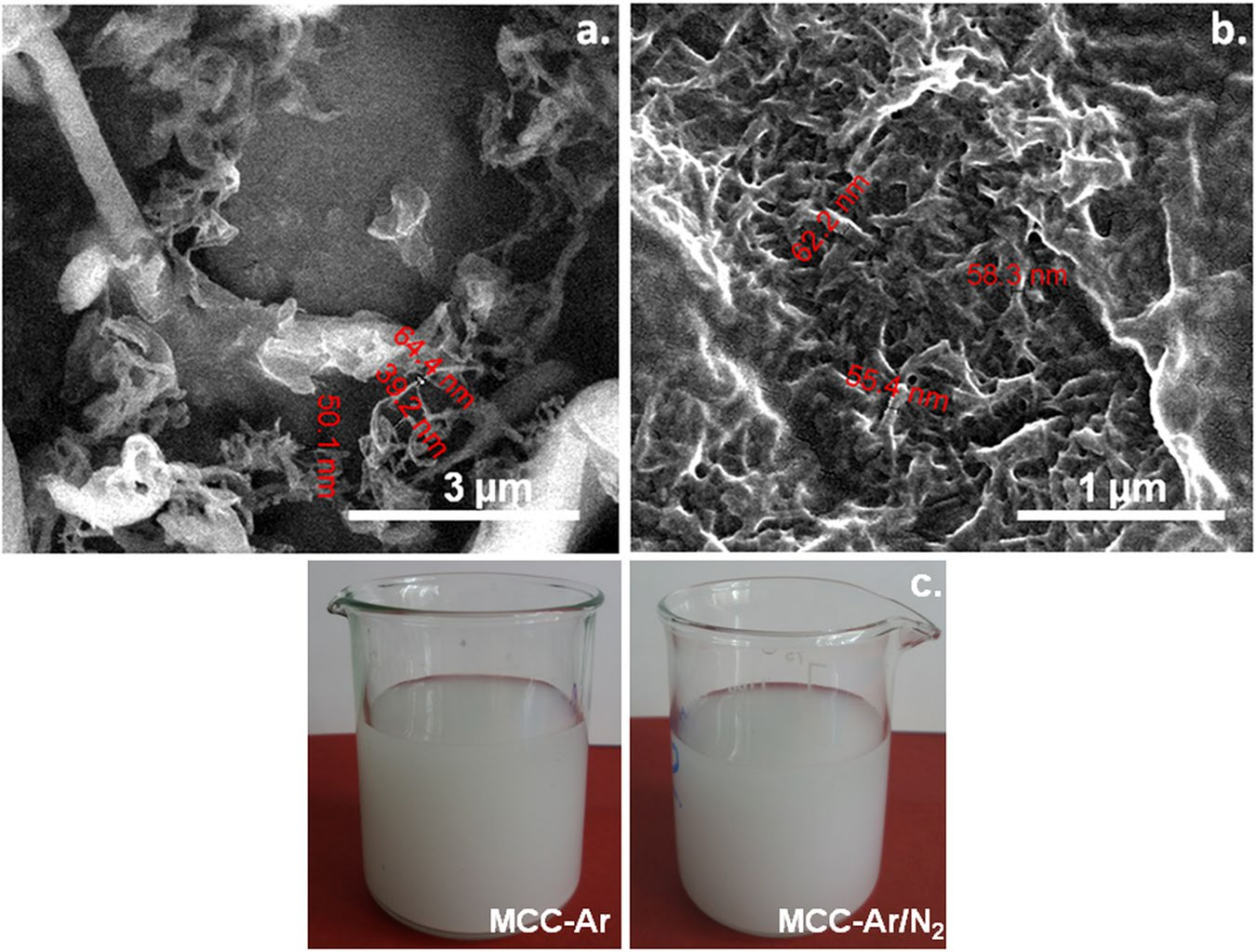
Detailed SEM images of Ar (**a**) and Ar/N_2_ (**b**) plasma treated MCC; (**c**) the supernantant is turbid for both Ar and Ar/N_2_ plasma treatments (24 hours after ultrasonication).

In contrast, Ar plasma – ultrasound treatment led to a strong defibrillation; both nanofibers, less than 100 nm in width and microfibers, 1–2 µm in width and the same length as the initial fibers were observed in Fig. 8c. In the detailed image from Fig. 9a many nanofibers of 40–60 nm in width and micrometric in length may be observed. Therefore, the Ar plasma treatment followed by ultrasonication led to a remarkable aspect ratio of the nanofibers which can reach 80. The defibrillation occured by the spliting of the fibers in thinner ones and the detachement of nanofibers from the surface of both initial and splitted fibers (Fig. 9a). The higher aspect ratio and the increased surface area of Ar treated cellulose fibers could be beneficial to the properties of the polymer composites obtained with these fibers. Both thinner fibers, of only 1 µm in width and nanofibers of 50–60 nm deposited as a network between microfibers were observed in the case of Ar/N_2_ treated MCC (Figs 8d and 9b). Microfibers with the width of about 1 µm were also observed in the case of Ar/O_2_ treatment and, especialy, after Ar-ACN treatment (Fig. 8f). It may be concluded that the defibrillation in thinner fibers and nanofibers will proceed with different intensity for SLP - ultrasound treated cellulose samples depending on the plasma treatment conditions.

Corroborating SEM observations with FTIR and XPS results, it was observed that Ar and Ar/N_2_ plasma treatments followed by ultrasonication led to an important defibrillation and, probably, to a higher nanofiber yield, while Ar/O_2_ and Ar-ACN led mostly to the surface functionalization of cellulose micro and nanofibers and to a more reduced defibrillation. Therefore, plasma treatment increased the efficiency of ultrasonication for the defibrillation of cellulose in certain conditions. Indeed, the photographs in Fig. 9c show the turbidity of the supernatant for the Ar and Ar/N_2_ plasma - ultrasound treated samples, 24 hours after the treatments, which is indicative of the presence of nanofibers^9^.

The nanofiber yield (Y) was estimated in the case of Ar and Ar/N_2_ plasma/ultrasound treated MCC relativ to MCC treated only by ultrasonication, Y being the fraction of cellulose that turn into nano. An increase of the yield from about 15% for ultrasonicated MCC to 49% for Ar plasma - ultrasonicated MCC and to 25% for Ar/N_2_ plasma and ultrasound treated MCC was obtained. Plasma treatment followed by ultrasonication is, thus, a green effective tool for the defibrillation of cellulose.

### Characterization of PHB composites with plasma treated cellulose

#### Thermal stability

**Figure 10:**
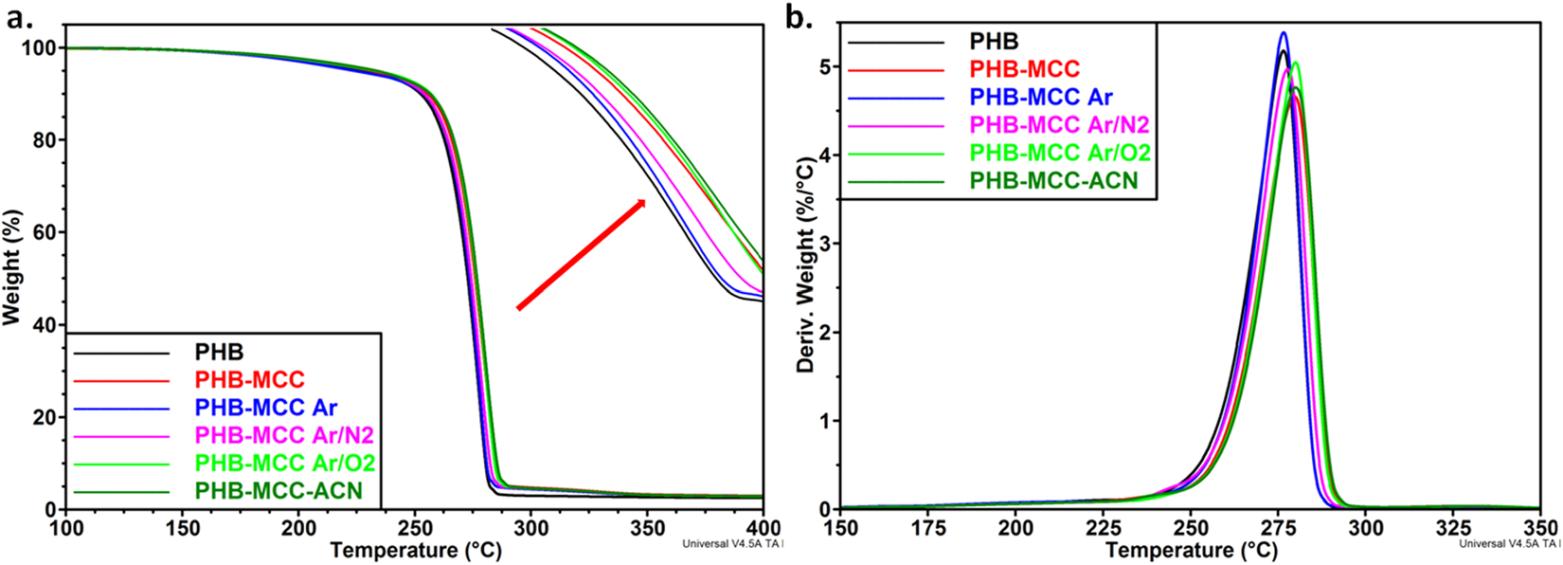
TGA and DTG curves for PHB-MCC composites containing plasma treated cellulose.

The degradation of PHB is a single step process with the onset degradation temperature at 263 °C and *T*_*max*_ at 277 °C (Fig. 10); this degradation step corresponds to the ester cleavage of PHB by β-elimination reaction^51^. Similar degradation process was noticed for composites with the difference that a shoulder appeared above 300 °C. The *T*_*max*_*(2)* of about 330 °C of this shoulder corresponds to the degradation of cellulose.

It is worth to mention that, although Ar/O_2_ and, especially, Ar-ACN treatments have led to the decrease of *T*_*max*_ value of cellulose, all the composites showed slightly higher thermal stability compared to pure PHB. A T_max_ value between 276 °C and 280 °C was obtained for the composites, regardless the treatment (Table S2 of the Supplementary Information). No important change of the thermal stability was reported for PHB composites containing nanocellulose treated by DBD plasma^22^. It results that the changes in thermal stability observed in the case of plasma treated MCC (Table S1) reflect only a surface activation, which may increase the compatibility with PHB. This may be verified by the mechanical characteristics of the composites with untreated and plasma treated cellulose.

#### Mechanical behavior

**Figure 11:**
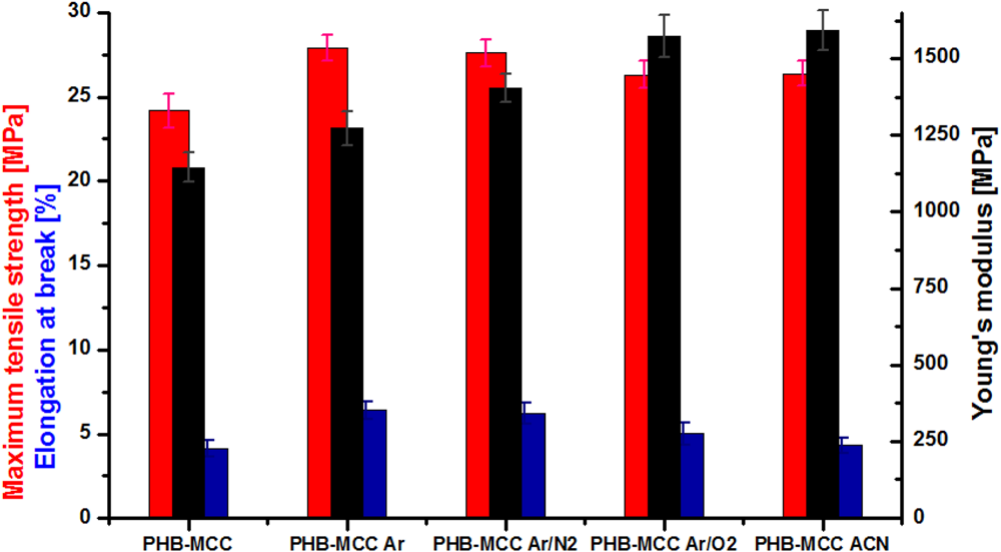
Mechanical characteristics of PHB composites with 2 wt% untreated and plasma treated MCC and standard deviation.

The mechanical properties of composites (maximum tensile strength, elongation at break and Young’s modulus) are shown in Fig. 11.

A slight increase of tensile strength and elongation at break was noticed in the case of PHB composites containing plasma treated cellulose. The highest increase (14–15%) in tensile strength was noticed for PHB composites containing plasma torch treated MCC in Ar or Ar/N_2_. Similar increase was reported for PHB nanocomposites with nanocellulose treated by DBD plasma in Ar, with or without ACN in the water suspension^22^. In contrast, a significant increase of Young’s modulus compared to PHB containing untreated MCC was observed. Thus, an increase of Young’s modulus by 40%, from 1145 MPa for the composite with untreated MCC to 1600 MPa for the composite containing Ar-ACN treated cellulose was noticed and a similar increase for the composite with Ar/O_2_ plasma treated cellulose. It may be assumed that Ar-ACN and Ar/O_2_ plasma treated cellulose are better reinforcing agents for PHB compared to untreated cellulose due to a better polymer - cellulose interface which results in better mechanical properties. This statement is supported by the results of FTIR and XPS analysis which showed the effect of Ar-ACN and Ar/O_2_ plasma treatments on the surface functionalization of cellulose. The surface activation of cellulose following plasma treatments may consist of more free OH groups (unbonded by hydrogen bonds) or the convergence of hydroxyl groups on the surface of cellulose in carbonyl or carboxyl groups or nitrogen containing groups. On the other hand, the intense defibrillation of cellulose promoted by the activation with Ar or Ar/N_2_ plasma treatments seem beneficial to the tensile strength and elongation at break of PHB composites containing these celluloses (Fig. 11). Indeed, a strong nanofibrillation was detected by SEM in the case of these treatments (Fig. 9). Overall, the treatment of cellulose by SLP led to better mechanical properties of PHB and it is proposed as a “clean” and efficient method to increase PHB performance in view of biomedical and engineering applications.

#### SEM investigation of fractured composites

**Figure 12:**
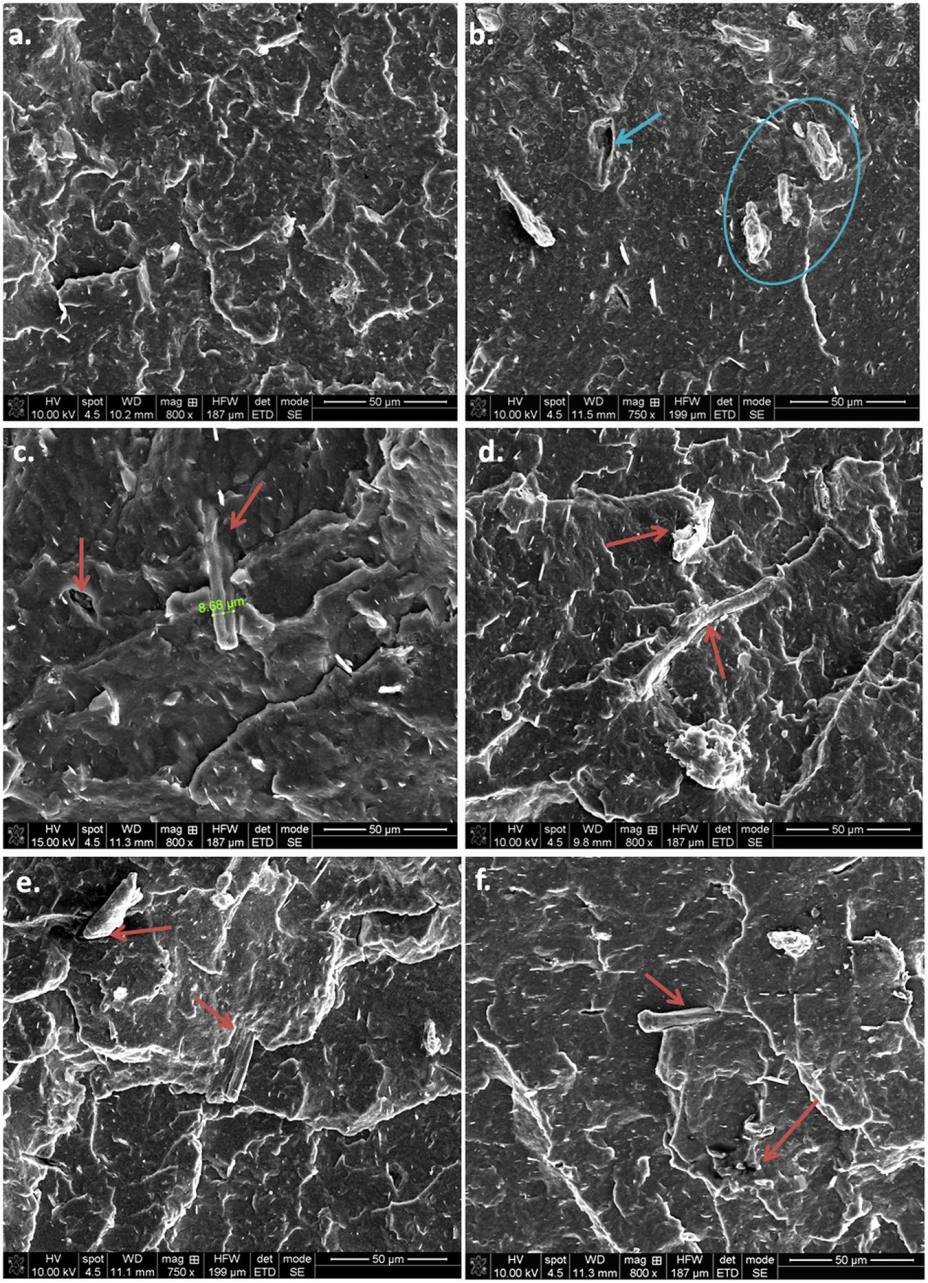
SEM images of fractured samples: PHB (**a**), PHB-MCC (**b**) and PHB-MCC Ar (**c**), PHB-MCC Ar/N_2_ (**d**), PHB-MCC Ar/O_2_ (**e**), PHB-MCC Ar-ACN (**f**).

**Figure 13:**
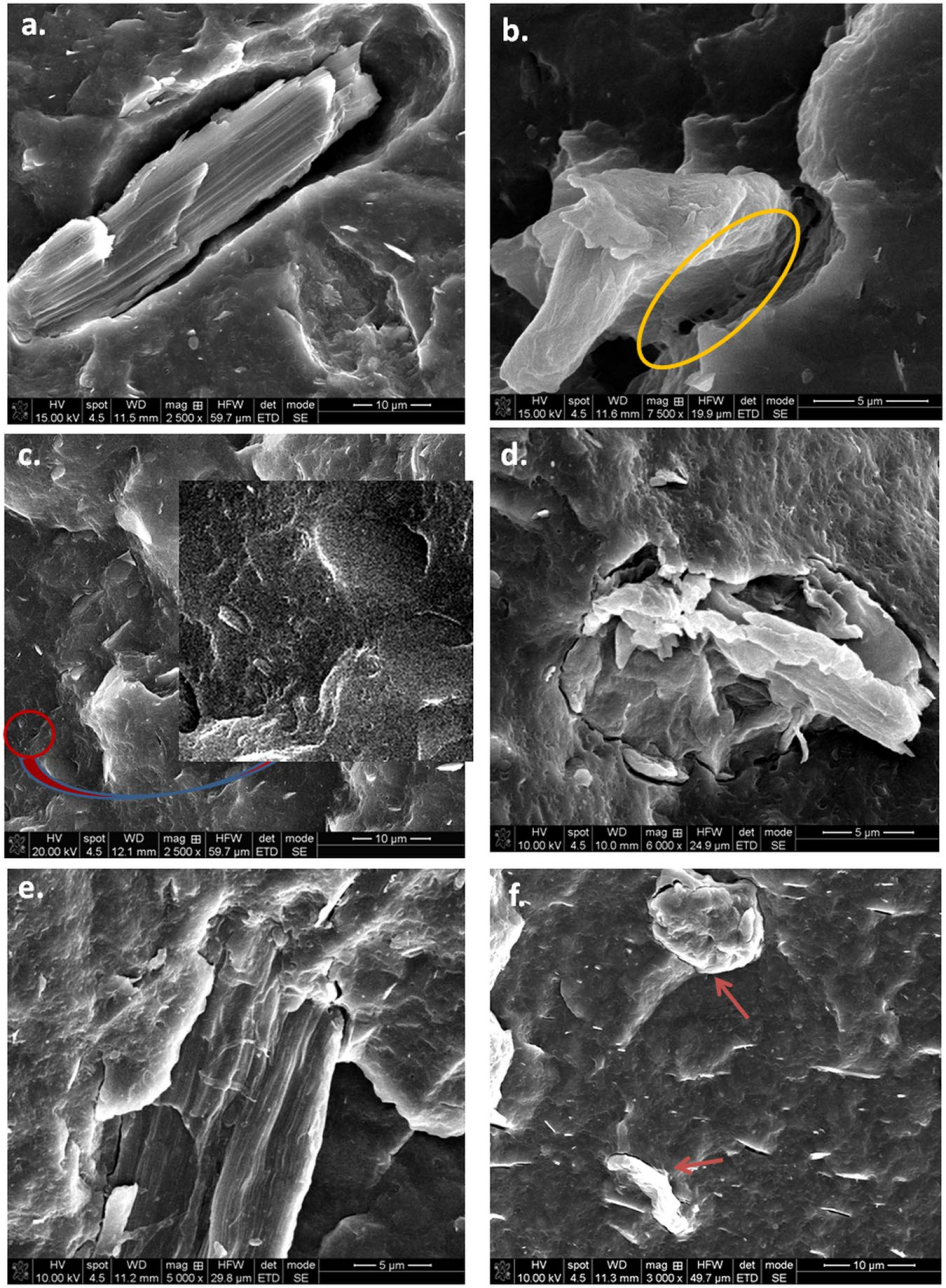
Interface in PHB-plasma treated MCC composites: PHB-MCC (**a**,**b**), PHB-MCC-Ar (**c**), PHB-MCC Ar/N_2_ (**d**), PHB-MCC- Ar/O_2_ (**e**) and PHB-MCC-Ar-ACN (**f**).

SEM images of fractured composites provide new insight into PHB-cellulose interface characteristics (Figs 12 and 13).

Large empty spaces, as that marked with arrow, and unbroken fibers with more than 20 μm in length out of the polymer surface may be seen in the SEM image of PHB-MCC (Fig. 12b), suggesting a poor polymer-filler interface. Higher magnification images show both large empty spaces around the fiber (Fig. 13a) and some punctual contacts between the cellulose fiber and PHB (Fig. 13b). At this high magnification, nanometric polymer strands which are bond to the cellulose fiber are visible in Fig. 13b.

Broken cellulose fibers were observed on the PHB-MCC Ar fracture, which shows that the polymer-Ar plasma treated cellulose interface is good enough to prevent the fiber detachment from the matrix. In the case of a strong interface, the fiber is broken. This shows an effective stress transfer between the fiber and the matrix^52^. Higher magnification image (Fig. 13c) reveals also nanometric cellulose fibers, similar to those observed by SEM in the case of Ar plasma treated MCC (Fig. 8c). Cellulose fibers covered by the polymer and a good interface may be also observed in the SEM images of PHB-MCC Ar/N_2_ and PHB-MCC Ar/O_2_ fractured samples (Figs 12d,e and 13d,e). Broken fibers well embedded in the polymer matrix were observed in the higher magnification images of PHB composites with Ar/N_2_, Ar/O_2_ -Ar and Ar- ACN plasma treated MCC (Fig. 13d–f). Thinner fibers, in the nanometric range, embedded in the polymer or on the surface of larger cellulose fibers were also noticed for these samples (Fig. 13d–f). To sum up, plasma treated MCC containing composites show better interface than PHB-pristine MCC and increased defibrillation, which explains the improved mechanical properties of these composites.

## Conclusions

In this study we report on a green, simple, and efficient method to defibrillate and functionalize cellulose. Plasma torch completely immersed in the cellulose suspensions followed by ultrasonication induces both defibrillation and surface functionalization of microcrystalline cellulose. We found that Ar/O_2_ and Ar-ACN plasma treatments have a higher influence on the surface structure and properties of MCC as compared to the Ar and Ar/N_2_ plasma treatments in conjunction with ultrasounds, which have a strong influence on the defibrillation of cellulose. The Ar plasma activation of cellulose applied prior to ultrasonication lead to a remarkable increase of the nanofibers yield. A slight decrease of the thermal stability of cellulose was induced by Ar-ACN or Ar/O_2_ plasma treatments. However, the thermal stability of the composites containing these celluloses as reinforcements showed an increase of the onset temperature of degradation. SEM images of fractured composites emphasized better polymer-cellulose interface in the case of plasma treated MCC containing composites and more advanced defibrillation. Therefore, the treatment of cellulose by submerged liquid plasma is highly encouraging for the improvement of the surface properties of cellulose and its reinforcing ability for biomedical and engineering applications.

## Electronic supplementary material

Supporting Information

## Data Availability

The data that support the findings of this study are available in the Supplementary Information and from the corresponding author upon reasonable request.
