# Supplementary material for: Cellulose defibrillation and functionalization by plasma in liquid treatment
Source: Sci Rep. 2018 Oct 19;8:15473. doi: 10.1038/s41598-018-33687-2 (PMC6195520; doi:10.1038/s41598-018-33687-2)
Supplement: Supplementary file 1 — Supporting Information [file 41598_2018_33687_MOESM1_ESM.docx]

***Supplementary information***

**Cellulose defibrillation and functionalization by plasma in liquid treatment**

Sorin Vizireanu^1^, Denis Mihaela Panaitescu^2,^*, Cristian Andi Nicolae^2^, Adriana Nicoleta Frone,^2^ Ioana Chiulan^2^, Maria Daniela Ionita^1^, Veronica Satulu^1^, Lavinia Gabriela Carpen^1^, Simona Petrescu^3^, Ruxandra Birjega^1^& Gheorghe Dinescu^1^

^1^ National Institute for Laser, Plasma and Radiation Physics, Atomistilor 409, Magurele-Bucharest, Ilfov 077125, Romania;

^2^ Polymer Department, National Institute for Research and Development in Chemistry and Petrochemistry, 202 Spl. Independentei, Bucharest 060021, Romania;

^3^ Institute of Physical Chemistry "Ilie Murgulescu", Romanian Academy of Sciences, 202 Spl. Independentei, Bucharest 060021, Romania;

*****Correspondence: [panaitescu@icechim.ro](mailto:panaitescu@icechim.ro)

**Table S1.** TGA results for plasma treated cellulose in different conditions (reactive gas, treatment duration and flow rate)

| **Samples** | ***T_on_***  **(°C)** | ***T_max_***  **(°C)** | ***R_500_***  **(%)** |
| --- | --- | --- | --- |
| MCC | 321.4 | 341.7 | 4.5 |
| MCC Ar-30min | 324.4 | 345.5 | 5.5 |
| MCC Ar-60min | 318.1 | 338.6 | 5.0 |
| MCC Ar/N_2_-30min | 321.6 | 341.9 | 5.0 |
| MCC Ar/N_2_-60min | 314.7 | 335.1 | 5.0 |
| MCC Ar-ACN | 300.3 | 325.7 | 12.3 |
| MCC Ar/O_2_-250 sccm | 318.1 | 339.5 | 5.8 |
| MCC Ar/O_2_-500 sccm | 311.0 | 332.3 | 6.1 |

**Table S2.** TGA characteristic temperatures of PHB composites containing untreated and plasma treated cellulose

| **Samples** | ***T_on_***  **(°C)** | ***T_max_***  **(°C)** |
| --- | --- | --- |
| PHB | 263.5 | 276.6 |
| PHB-MCC | 266.3 | 279.9 |
| PHB-MCC Ar | 265.4 | 276.7 |
| PHB-MCC Ar/N_2_ | 265.3 | 277.8 |
| PHB-NC Ar/O_2_ | 267.4 | 280.1 |
| PHB-MCC Ar-ACN | 267.4 | 280.0 |
